# Stress-mediated aggregation of disease-associated proteins in amyloid bodies

**DOI:** 10.1038/s41598-023-41712-2

**Published:** 2023-09-02

**Authors:** Sahil Chandhok, Lionel Pereira, Evgenia A. Momchilova, Dane Marijan, Richard Zapf, Emma Lacroix, Avneet Kaur, Shayan Keymanesh, Charles Krieger, Timothy E. Audas

**Affiliations:** 1 Department of Molecular Biology and Biochemistry, Simon Fraser University, 8888 University Drive, €, BC V5A 1S6 Canada; 2 Centre for Cell Biology, Development, and Disease, Simon Fraser University, 8888 University Drive, Burnaby, BC V5A 1S6 Canada; 3 Department of Biomedical Physiology and Kinesiology, Simon Fraser University, 8888 University Drive, Burnaby, BC V5A 1S6 Canada

**Keywords:** Protein aggregation, Nucleolus

## Abstract

The formation of protein aggregates is a hallmark of many neurodegenerative diseases and systemic amyloidoses. These disorders are associated with the fibrillation of a variety of proteins/peptides, which ultimately leads to cell toxicity and tissue damage. Understanding how amyloid aggregation occurs and developing compounds that impair this process is a major challenge in the health science community. Here, we demonstrate that pathogenic proteins associated with Alzheimer’s disease, diabetes, AL/AA amyloidosis, and amyotrophic lateral sclerosis can aggregate within stress-inducible physiological amyloid-based structures, termed amyloid bodies (A-bodies). Using a limited collection of small molecule inhibitors, we found that diclofenac could repress amyloid aggregation of the β-amyloid (1–42) in a cellular setting, despite having no effect in the classic Thioflavin T (ThT) in vitro fibrillation assay. Mapping the mechanism of the diclofenac-mediated repression indicated that dysregulation of cyclooxygenases and the prostaglandin synthesis pathway was potentially responsible for this effect. Together, this work suggests that the A-body machinery may be linked to a subset of pathological amyloidosis, and highlights the utility of this model system in the identification of new small molecules that could treat these debilitating diseases.

## Introduction

Amyloids are proteinaceous aggregates that have been associated with a wide variety of systemic and neurological pathologies^1^. These insoluble structures stain positively with amyloidophilic dyes (Congo-red and Thioflavin T:ThT)^1^, and display a high degree of structural stability due to tight stacking of polypeptides into “rope-like” fibers^2–4^. The transition of proteins from soluble monomers into insoluble aggregates takes place via a nucleation-dependent growth mechanism^1,4^. Here, the duration of the lag phase is determined by the time required to generate critical nuclei, with environmental variables such as temperature, pH, salt and protein concentrations having a strong influence on the reaction kinetics^5–7^. These amyloid oligomers typically have high free energy, and their presence initiates rapid polymerization of fibrils^1,4,5^.

Currently, amyloid aggregation has been linked to over 25 human disorders, with pathological amyloid aggregates being deposited in both, intracellular and extracellular spaces^1,8^. These structures are believed to damage host tissue in a variety of ways, including the perturbation of cellular membrane^9^, disruption of proteasomal function^10^, production of reactive oxygen species^11^, or inhibition of autophagy^12^. The development of disease-associated aggregates has been attributed to abnormal cleavage of amyloidogenic peptides (e.g. β-amyloid (1–42) in Alzheimer’s disease); over-expression of intrinsically disordered proteins (e.g. amylin in type 2 diabetes); and the introduction of amyloidogenic mutations into wild-type amino acid sequences (e.g. SOD1 in amyotrophic lateral sclerosis: ALS)^13–16^. Also, factors such as ischemic stroke, hyperthermia, and inflammation appear to influence the rate of plaque deposition^17–19^, suggesting that both genetic and environmental features play an important role in disease etiology. Despite the historical prominence of amyloids in pathological settings, novel physiological functions for this protein conformation have emerged over the past two decades^20^. Functional amyloid aggregates are known to play a central role in bacterial biofilms^21^, fungal reproduction^22^, *Xenopus* oocyte development^23^, and the regulation of various mammalian pathways^24–26^.

Recently, a well conserved eukaryotic subnuclear domain, termed the amyloid body (A-body), was found to sequester proteins in an amyloid-like state in response to environmental stressors (heat shock, hypoxia/acidosis, and transcriptional/proteotoxic stress)^27–30^. These structures share many of the biophysical characteristics ascribed to pathological protein deposits, as they are dense, proteinase K-resistant, insoluble aggregates that possess immobile molecular constituents and a strong affinity for the amyloidophilic dyes Congo red and Thioflavin S^27^. However, unlike their disease-associated counterparts, A-body biogenesis is a rapid and reversible process that generates non-toxic physiological structures^27,31^. Upon stimulation, these biomolecular condensates displace nucleoli by sequestering a large and heterogeneous family of cellular proteins^27,29^. The distinct stressors aggregate both universal and unique molecular residents^29^, potentially enhancing survival under harsh environmental conditions by tailoring a metabolic response to each cellular insult. Proteins found in A-bodies under all conditions tested, such as cell division cycle protein 73 (CDC73), represent universal A-body residents, and are useful marker molecules of these subnuclear foci^29^. The presence of unique constituents, such as flap endonuclease 1 (heat shock), DNA methyltransferase 1 (hypoxia/acidosis) and anaphase-promoting complex subunit 2 (transcriptional/proteotoxic stress) is particularly fascinating^29^. This observation suggests that aggregation of a subset of proteins is catalyzed by stress-specific machinery, which mediates the recruitment and amyloid conversion of these proteins only under specific environmental conditions. A-bodies are also shown to be capable of recruiting and aggregating the Alzheimer’s disease peptide β-amyloid^27^, suggesting that pathological amyloidogenesis may be linked to the dysregulation of processes associated with A-body formation. In this study, we assess the ability of various disease-associated proteins to aggregate within the A-bodies. We also explore the cellular pathways and mechanisms regulating this aggregation event. This data could provide useful insights into the origins of several amyloidogenic disorders and a framework for the development of a new screen platform for therapeutics.

## Results

### Amyloid disease-associated proteins aggregate within A-bodies

**Figure 1:**
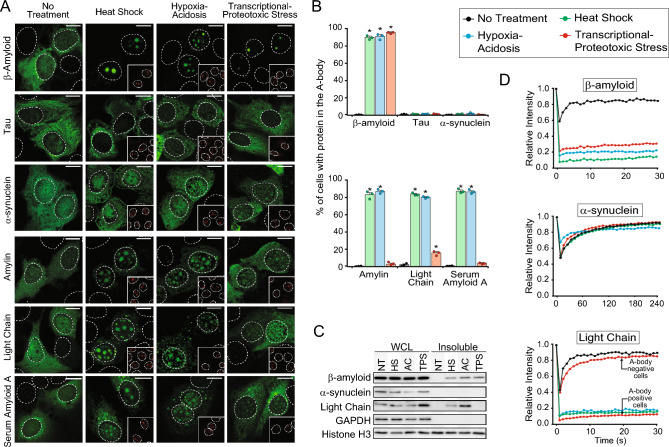
Disease-associated proteins can aggregate within physiological amyloids under specific stimuli (**A**) MCF-7 cells transfected with plasmids encoding β-amyloid (1–42)-GFP, Tau-GFP, α-synuclein-GFP, amylin-GFP, immunoglobulin light chain-GFP, and Serum Amyloid A-GFP were exposed to heat shock (HS), hypoxia/acidotic (AC) or transcriptional/proteotoxic stress (TPS) conditions for 2 h. Endogenous CDC73 was immuno-stained (red-inset) as an A-body control. (**B**) The percentage of MCF-7 cells with the constructs described above targeted to the A-bodies was calculated under each environmental condition. Values represent means ± s.e.m (n = 3), with *p ≤ 0.05. (**C**) MCF-7 cells expressing β-amyloid (1–42)-GFP, α-synuclein-GFP, or immunoglobulin light chain-GFP were treated as indicated prior to extraction. Whole cell lysates (WCL) and insoluble fractions were extracted and run on a western blot. GAPDH (soluble) and Histone H3 (insoluble) were used as loading controls. (**D**) Quantification of the fluorescence recovery after photobleaching for β-amyloid (1–42)-GFP, α-synuclein-GFP, and immunoglobulin light chain-GFP in treated MCF-7 cells. A-bodies were bleached, where present (n = 10). Dashed circles represent nuclei and white bars: 10 µm.

A-bodies represent a physiological site of protein aggregation, where cellular factors and local environmental conditions have created a setting that is conducive to the residents proteins adopting an amyloid-like conformation^27^. Here, we assess the ability of a variety of fibrillation-prone amyloidosis-associated proteins, to aggregate within A-bodies. Under normal growth conditions, GFP-tagged β-amyloid (1–42) (Alzheimer’s disease), Tau (Alzheimer’s/Parkinson’s disease), α-synuclein (Parkinson’s disease), amylin (type II diabetes), immunoglobin light-chain (AL amyloidosis), and serum amyloid A (AA amyloidosis) can be found throughout transfected cells (Fig. 1A,B), possessing highly soluble and mobile properties (Fig. 1C,D). However, in response to environmental stressors only a subset of the tested disease-associated proteins translocate to the nucleus, co-localize with the A-body marker molecule CDC73^29^ (Fig. 1A,B), and adopt the hallmark insoluble and immobile characteristics of these amyloid-like structures (Fig. 1C,D). It is interesting to note that like physiological A-body constituents^29^, pathological proteins also possess stress-specific targeting properties. For example, β-amyloid (1–42) was found to aggregate under all known A-body-inducing stimuli, while serum amyloid A and amylin primarily localized to A-bodies under heat shock and hypoxic/acidotic conditions (Fig. 1A,B). Immunoglobulin light-chain was recruited and immobilized within the A-bodies of heat shock-, hypoxia/acidosis-, and a sub-population of transcriptional/proteotoxic stress (TPS)-treated cells (Fig. 1A–D). However, the un-sequestered proteins maintained a mobile profile (Fig. 1D-bottom panel), highlighting that molecules found within these structures adopt the amyloid-like properties of this subnuclear domain, while the untargeted proteins remain highly mobile under the same environmental conditions.

**Figure 2:**
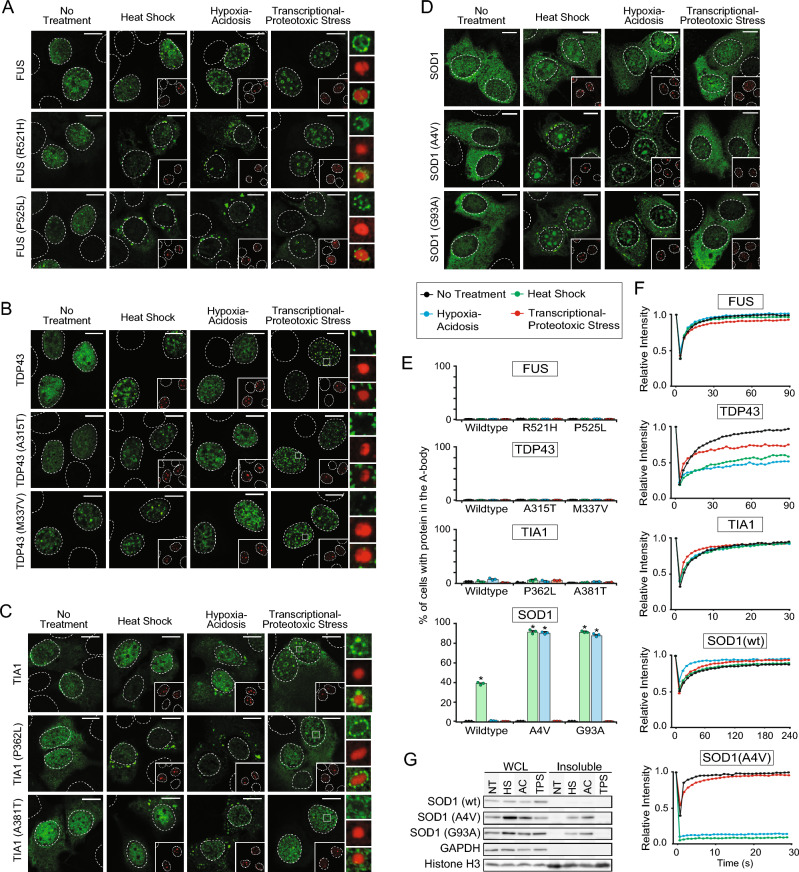
Targeting of wildtype and mutant ALS-associated proteins to A-bodies. (**A–D**) GFP fusion proteins for (**A**) FUS, FUS(R521H), FUS(P525L), (**B**) TDP43, TDP43(A315T), TDP43(M337V), (**C**) TIA1, TIA1(P362L), TIA1(A381T) or (**D**) SOD1-GFP, SOD1(A4V)-GFP or SOD1(G93A)-GFP were expressed in MCF-7 exposed to heat shock (HS), hypoxic/acidotic (AC) or transcriptional/proteotoxic stress (TPS) conditions for 2 h. mCherry-tagged CDC73 was co-expressed as an A-body control (red-inset). Indicated regions of TPS-treated cells (white squares) were expanded on the side, with merged image shown on the bottom. (**E**) The proportion of cells with FUS, TDP43, Tia1, or SOD1 wild-type and mutant constructs targeted to A-bodies was determined. Values represent means ± s.e.m (n = 3), with *p ≤ 0.05. (**F**) Quantification of the fluorescence recovery after photobleaching for wild-type FUS-GFP, TDP-43-GFP, TIA1-GFP, SOD1-GFP, and SOD1(A4V)-GFP in MCF-7 cells treated as indicated (n = 10). (**G**) MCF-7 cells expressing SOD1-GFP, SOD1(A4V)-GFP, or SOD1(G93A)-GFP were treated prior to extraction, fractionation and western blotting. GAPDH and Histone H3 were used as solubility controls. Dashed circles represent nuclei and white bars: 10 µm.

Many of the proteins described above aggregate with wild-type sequences, however, the pathology of other disease-associated proteins can be tied to fibrillation-promoting mutations. We next considered fused in sarcoma (FUS), TAR DNA-binding protein 43 (TDP-43), T-cell-restricted intracellular antigen-1 (TIA1), and superoxide dismutase 1 (SOD1), which often contain missense substitutions in ALS patients. Expression of wild-type FUS, TDP-43, and TIA1 in cells exposed to A-body-inducing stimuli failed to result in significant A-body targeting or protein immobilization (Fig. 2A–F), though wild-type SOD1 was weakly sequestered in a subset of heat shock-treated cells (Fig. 2E). Therefore, we used site-directed mutagenesis to introduce two common pathological amino acid substitutions into each protein and re-assessed their A-body targeting/aggregation potential. Familial ALS-associated SOD1 mutants (A4V and G93A)^13,15^ were efficiently recruited in hypoxia/acidosis- and heat shock-induced A-bodies (Fig. 2D,E), whereas none of the disease-associated FUS (F521H and P525L), TDP-43 (A315T and M337V), or TIA1 (P362L and A381T) mutations altered the affinity of these proteins for this subnuclear domain (Fig. 2A–C,E). The mutant SOD1 proteins also adopted the characteristic insoluble and immobile properties seen by other A-body constituents, demonstrating that protein aggregation was occurring under these conditions (Fig. 2F,G). FUS and TIA1 mutant constructs were targeted to cytoplasmic foci during heat shock/acidotic treatments and peri-A-body caps during TPS exposure (Fig. 2A,C). These results align with published reports demonstrating the presence of these proteins in cytoplasmic stress granules and nucleolar caps upon exposure to comparable stimuli^32–35^. Together, these data demonstrate that a subset of pathological proteins can utilize stress-specific A-body assembly machinery to rapidly induce their aggregation, suggesting that components of this physiological pathway may be associated with the disease etiology of a subgroup of human amyloidoses.

### A-body aggregation of pathological proteins can be pharmacologically impaired

**Figure 3:**
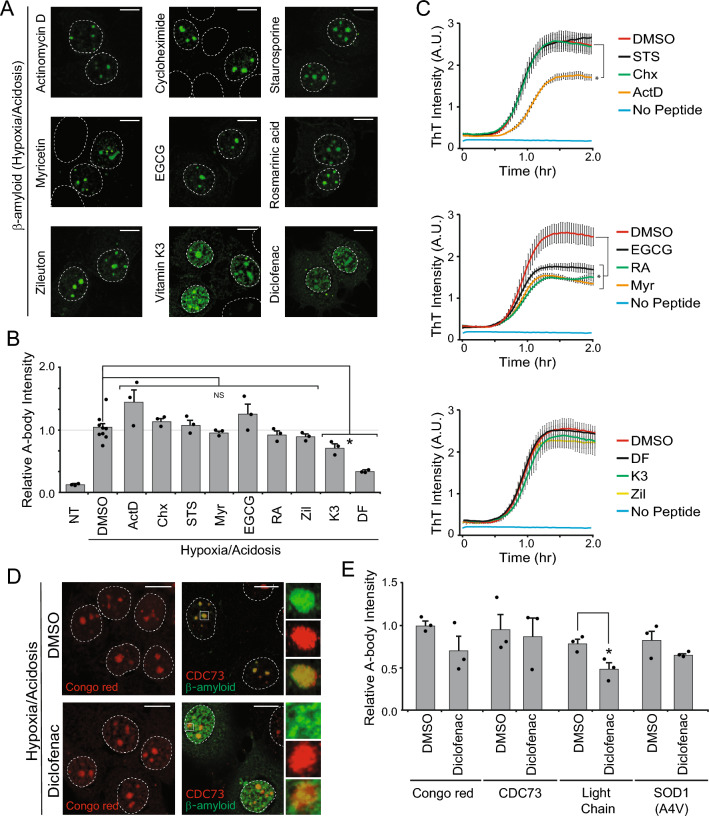
Pharmacological inhibition of the targeting of pathological protein to A-bodies. (**A**) β-amyloid (1–42)-GFP was visualized in MCF-7 cells pre-treated for 1 h with DMSO, 4 µM Actinomycin D (ActD), 50 µg/mL Cycloheximide (Chx), 100 nM Staurosporine (STS), 50 µM Myricetin (Myr), 50 µM Epigallocatechin gallate (EGCG), 50 µM Rosmarinic acid (RA), 100 µM Zileuton (Zil), 25 µM Vitamin K3 (K3), or 100 µM Diclofenac (DF) prior to a 2-h hypoxia/acidosis exposure. (**B**) A-body targeting efficiency was calculated for the samples treated above. Relative A-body intensity was calculated by measuring the intensity of the A-body signal relative to the background nucleoplasmic fluorescence. (**C**) A Thioflavin T assay was used to measure in vitro aggregation kinetics for the β-amyloid (1–42) peptide over a 2-h time frame in the presence of 2 µM ActD, 25 µg/ml Chx, 50 nM STS, 25 µM Myr, 25 µM EGCG, 25 µM RA, 50 µM Zil, 12.5 µM K3, or 50 µM DF. Arbitrary units (A.U.). (**D**) Hypoxic/acidotic MCF-7 cells pre-treated with DMSO or 100 µM Diclofenac were stained with the Congo red or antibodies detecting the endogenous A-body marker CDC73 in β-amyloid (1–42)-GFP transfected cells. Indicated regions (white squares) were expanded beside, with merged image shown on the bottom. (**E**) Quantification of Congo red, endogenous CDC73, and transfected β-amyloid (1–42)-GFP, immunoglobulin light chain-GFP, or SOD1(A4V)-GFP A-body targeting under hypoxic/acidotic conditions with either a DMSO or Diclofenac pre-treatment. Dashed circles represent nuclei and white bars: 10 µm. Values represent means ± s.e.m (n ≥ 3), with *p ≤ 0.05.

As A-bodies can capture known pathological proteins (Figs. 1, 2), we sought to uncover the molecular pathways regulating this process and determine whether this could be chemically impaired within a cellular setting. We focused on hypoxia/acidosis as the A-body-inducing stimulant, because the low pH (6.0) and oxygen (1%) environment we use mimics the effects of reduced blood flow seen in a stroke setting, which has been shown to promote Alzheimer’s disease pathogenesis^36–38^. Using an established method for quantifying the efficiency of A-body targeting^28^, we first assessed whether broad inhibitors of transcription (actinomycin D), translation (cycloheximide), and kinase signaling (staurosporine) were capable of having an impact on β-amyloid (1–42) targeting (Fig. 3A,B). Our results indicate that repressing de novo gene expression, protein synthesis, and kinase activity had no significant impact on A-body recruitment of β-amyloid (1–42), suggesting that cellular factors required for amyloid aggregation are likely present prior to stress exposure. Moving forward, we selected a panel of small molecules that were either shown to directly impair in vitro fibrillation (myricetin, epigallocatechin-gallate: EGCG, and rosmarinic acid)^39–43^ or epidemiologically reduce the risk of neurological disease (Zileuton, Vitamin K, and Diclofenac)^44–46^. Under the conditions tested, the in vitro fibrillation inhibitors all failed to repress A-body recruitment of β-amyloid (1–42) in a cellular setting (Fig. 3A,B), however, two of the chemicals associated with a diminished risk of neurological disease (Vitamin K3 and Diclofenac) significantly impaired targeting to A-bodies under hypoxic/acidotic conditions (Fig. 3A,B). To compare the results from a cellular setting to the established ThT in vitro fibrillation assay, we screened all nine compounds using this classic tube-based approach. Interestingly, there was no overlap in the small molecules that impaired aggregation in the cell-based (Fig. 3B) and in vitro Thioflavin T-based (Fig. 3C) assays. Actinomycin D and the fibrillation inhibitors myricetin, EGCG, and rosmarinic acid re-capitulated their previously observed in vitro repression of β-amyloid (1–42) aggregation (Fig. 3C: top and middle panel)^39–43^, while the cellular inhibitors of A-body recruitment (vitamin K3 and diclofenac) had no significant effect in the tube-based assay (Fig. 3C: bottom panel). These findings highlight the potential value of using a cellular model to assess aggregation, as in vitro inhibitors may not be effective under physiological conditions.

Next, we wanted to determine whether the strong diclofenac-mediated impairment was specific to the β-amyloid (1–42) peptide, or if it had a broad inhibitory effect on the targeting of physiological and pathological proteins. The gross formation of A-bodies was assessed using the amyloidophilic dye Congo red and the cellular marker molecule CDC73, while the targeting efficiency of additional disease-associated protein (SOD [A4V] and immunoglobulin light chain) was also determined. Our results demonstrated that A-body formation remained largely intact in diclofenac-treated cells, as the Congo red signal and CDC73 targeting efficiency were not statistically reduced (Fig. 3D,E). Like β-amyloid (1–42), the A-body targeting of immunoglobulin light chain was impaired, though recruitment of the ALS mutant SOD1(A4V) was not statistically different in the presence or absence of diclofenac (Fig. 3E). Together, these results suggest that the diclofenac-mediated effect on β-amyloid (1–42) and immunoglobulin light chain A-body targeting may be indirect, through the modulation of a cellular pathway that has some specificity towards these disease-associated proteins.

### The Cyclooxygenase (COX) pathway regulates recruitment of β-amyloid to A-bodies

**Figure 4:**
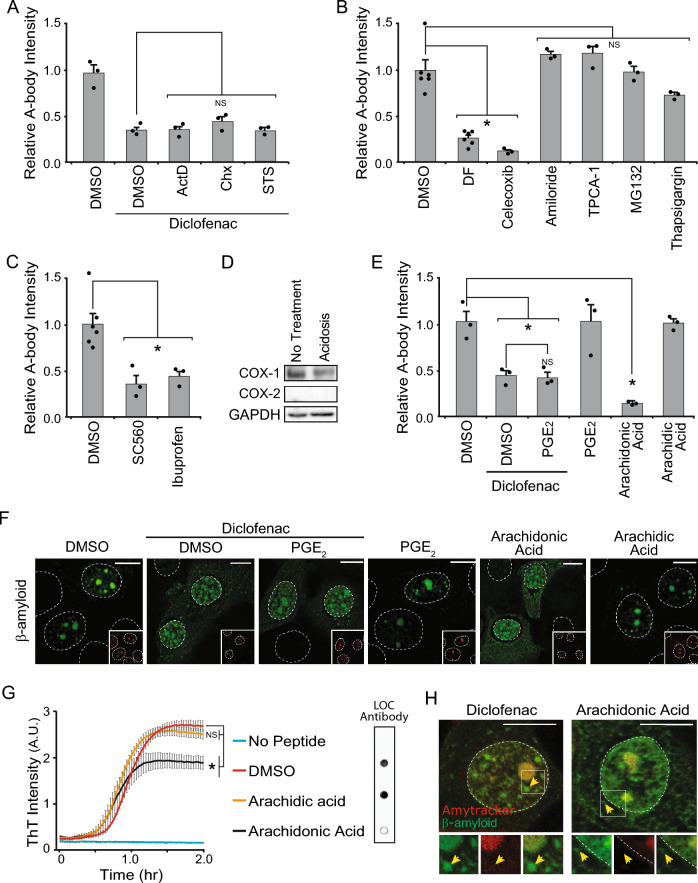
Dysregulation of cyclooxygenase (COX) and unsaturated fatty acids can impair β-amyloid (1–42) aggregation. (**A**) MCF-7 cells expressing β-amyloid (1–42)-GFP were co-pre-treated with Diclofenac and ActD, Chx, or STS prior to hypoxia/acidosis exposure. Relative A-body intensity was quantified. (**B**) β-amyloid (1–42)-GFP targeting under hypoxic/acidotic conditions was quantitatively assessed in cells pre-treated with diclofenac, 50 µM celecoxib, 300 µM amiloride, 8 µM MG132, 20 µM Thapsigargin, or 20 µM TPCA-1. (**C**) COX inhibitors SC560 (25 µM) or Ibuprofen (200 µM) were used as a pre-treatment for β-amyloid (1–42)-GFP transfected MCF-7 cells exposed to hypoxia/acidosis. A-body targeting efficiency was quantified. (**D**) Protein lysates for untreated and hypoxic/acidotic cells were harvested to detect COX1 and COX2 expression. GAPDH was used as a loading control. (**E–F**) Hypoxic/acidotic MCF-7 cells were treated with 100 µM Diclofenac, 1 µM Prostaglandin E2 (PGE2), 100 µM Arachidonic Acid, or 100 µM Arachidic Acid. A-body targeting was (**D**) quantified and (**E**) representative images were captured. Endogenous CDC73 was immuno-stained (red-inset) as an A-body control. (**G**) β-amyloid (1–42) peptides were incubated with arachidonic (50 µM) or arachidic (50 µM) fatty acids. In vitro fibrillation was assessed over a 2-h time frame with Thioflavin T (left panel). Peptide samples aggregated and incubated with the indicated compounds, were also dot blotted to assess fibrillation using the anti-amyloid fibrils LOC antibody (right panel). (**H**) β-amyloid (1–42)-GFP-expressing MCF-7 cells were pre-treated for 1 h with diclofenac (100 µM) or arachidonic acid (100 µM). After a two-hour hypoxia/acidosis treatment, Amytracker dye (red) was added to the media of live cells and images were captured. Indicated regions (white squares) were expanded below, with merged image shown on the right. Dashed circles represent nuclei and white bars: 10 µm. Values represent means ± s.e.m (n ≥ 3), with *p ≤ 0.05.

To assess the putative mechanism of this diclofenac-mediated impairment, we co-treated cells with diclofenac and inhibitors of transcription, translation, or kinase activity. The results from this experiment suggest that the diclofenac-induced targeting impairment is not dependent upon the expression of new gene products or kinase-dependent signalling cascades (Fig. 4A). As a non-steroidal anti-inflammatory drug, diclofenac has several on- and off-target effects. It has been shown to impair cyclooxygenases (COX), acid-sensing ion channels, IKK-2, and the proteosome, while also inducing ER stress^47–51^. Thus, we tested inhibitors for each of these proteins/pathways and assessed whether they were also capable of repressing β-amyloid aggregation. While the ER-stressor (Thapsigargin), and inhibitors of acid-sensing ion channels (amiloride), IKK-2 (TPCA-1), and the proteosome (MG132) had no effect, the COX inhibitor (celecoxib) mimicked the repression observed by diclofenac treatment (Fig. 4B). Two additional COX inhibitors (SC560 and ibuprofen) also significantly repressed β-amyloid (1–42) targeting to A-bodies (Fig. 4C), further highlighting the role of prostaglandin synthases in protein aggregation. In MCF-7 cells, only COX1 is expressed at detectable levels (Fig. 4D), and the protein is responsible for converting the unsaturated fatty acid arachidonic acid into prostaglandins^52^. This suggests that either elevated arachidonic acid levels or a deficiency of prostaglandins may be linked to the blocking of β-amyloid (1–42) recruitment. The addition of prostaglandin E2 (PGE_2_) to diclofenac-treated cells did not rescue A-body targeting, however, the application of arachidonic acid efficiently impaired β-amyloid (1–42) localization (Fig. 4E,F). This fatty acid also repressed fibrillation in a ThT and α-amyloid fibril dot-blot assays (Fig. 4G)^53^, while a lipid of similar length that is not utilized by prostaglandin synthases (arachidic acid), had no effect in the in vitro or cellular settings (Fig. 4E,F). Our observations indicate that treatment of hypoxic/acidotic cells with diclofenac and arachidonic acid leads to increased cytoplasmic and nuclear accumulation of β-amyloid (1–42) (Fig. 4F). Thus, we assessed whether these β-amyloid (1–42)-containing structures possessed the same amyloid-like characteristics as the A-body population using Amytracker, a live-cell amyloidogenic dye. As expected, A-bodies stained positively with Amytracker, but other nuclear and cytoplasmic β-amyloid (1–42)-containing structures were not amyloid-like in nature (Fig. 4H). Together, this data suggests that diclofenac can regulate β-amyloid (1–42) aggregation, potentially through an increase in unsaturated fatty acids levels.

## Discussion

The cellular processes involved in amyloidogenic diseases are not well characterized, so to explore these pathways we used stress-inducible functional amyloids as a model system. In this report, we demonstrate that a subset of proteins associated with neurodegenerative and systemic amyloid disorders aggregate within A-bodies (Fig. 1), suggesting common mechanisms exist between physiological and pathological amyloid-based aggregation. Our observations indicate that components of this physiological pathway could contribute to disease progression, perhaps by the dysregulation of factors associated with A-body formation. However, the participation of the A-body pathway does not appear to be a universal characteristic of pathological amyloidogenesis, indicating that there is potentially a divergence in the pathogenic origins of the various aggregation-based disorders. For example, FUS and TIA1 mutants were not targeted to A-bodies under any of the stress treatments examined (Fig. 2), but did aggregate in what appear to be cytoplasmic stress granules^32,34,54,55^. Thus, the distinct characteristics and regulators of these two membrane-less organelles could be linked to different pathological conditions, and further study may lead to a stratification of the amyloidogenic disorders based on their phase transition pathway of origin.

These observations also provide an exciting opportunity for translational research, as this work could be developed into a new cell-based approach for the identification or validation of compounds that impair pathological protein aggregation. Our small-scale pharmacological analysis of β-amyloid (1–42) inhibitors (Fig. 3) could serve as a proof-of-principle that this system may have utility as a new drug discovery platform. Here, we found that compounds known to impair amyloid fibrillation of disease-associated proteins in vitro^39–43,56,57^ had no detectable effect on aggregation in a cellular setting. We suspect this divergence may be attributed to factors including: short biological half-life or low membrane permeability, as myricetin, EGCG and rosmarinic acid are polar compounds. Additionally, the large GFP-tag associated with the pathological proteins in our cell-based setting could also alter the aggregation properties of a protein, leading to false negatives. However, what is unmistakably true is that the sole use of in vitro approaches would exclude the detection of small molecules that indirectly regulate amyloid aggregation. The anti-inflammatory drug diclofenac may be a prime example of this, as it has been linked to reduced risk of Alzheimer’s disease^44,58^ and shows a strong impairment of A-body-mediated β-amyloid (1–42) aggregation in this cell-based assay, however, it failed to supress in vitro fibrillation. Thus, a combinatory approach using in silico^59^, in vitro^60,61^, bacterial^62^, and model organism-based^63,64^ screening platforms along with rapid validation in this A-body model may provide a holistic approach to optimize drug candidate selection. Furthermore, we envision this model could be refined to use cell lines that more closely resemble the site of plaque formation or examining the aggregation potential of additional pathological proteins that have been linked to the various amyloidogenic disorders. Overall, this system clearly fills an underserviced niche, as the creation of drug discovery models for the study of amyloidogenic diseases is a pressing need in the therapeutic community^65^.

Despite the extracellular localization of some disease-associated plaques (e.g., β-amyloid (1–42) and amylin), the rapidity with which these peptides convert from a diffuse and soluble molecule (no treatment) to an immobile and insoluble aggregate (stress treatment) within the cell demonstrates that environmental conditions or aberrant exposure of these peptides to A-body regulators may play a role in transitioning to an amyloid state during disease progression. We envision that localized cell death may release these cellular factors into the extracellular milieu, which could decrease the nucleation lag-phase of proximal pathological peptides and enhance fibrillation kinetics. Thus, a better understanding of cellular factors regulating this stress-response pathway may identify novel genetic risk factors associated with amyloid diseases, while exploitation of this cell-based system for drug discovery could produce new therapeutics. Together, this work makes A-body biology a new frontier for human health research.

## Methods

### Cell culture, plasmids, treatments, and transfections

MCF-7 cells (ATCC) were maintained in Dulbecco’s Modified Eagle’s Medium (DMEM) (high glucose) which was supplemented with 10% (v/v) fetal bovine serum and 1% (v/v) penicillin–streptomycin, which were incubated at 37 °C and 5% carbon dioxide. Plasmids expressing β-amyloid (1–42)-GFP and TIA1-GFP were previously described ^27^, and Tau, α-synuclein, amylin, serum amyloid A, SOD1, FUS, and TDP43 cDNA were generated by RT-PCR from MCF-7 RNA and cloned into the plasmid pEGFP-C1 (BD Biosciences). SOD1(A4V), SOD1(G93A), FUS(R521H), FUS(P525), TDP43(A315T), TDP43(M337V), TIA1(P362L), and TIA1(A381T) mutants were generated by site-directed mutagenesis of the wildtype cDNA. The immunoglobulin light chain variable domain cDNA was synthesized as a gBlock gene fragment (Integrated DNA Technologies) encoding the AL-09 variant of the $\kappa$I O18:O8 germline sequence ^66^. Cells were transfected with polyethyleneimine (ThermoFisher Scientific) 24 h prior to all treatments, and either exposed to heat shock (43 °C) or hypoxia/acidosis (pH 6.0 media at 1% oxygen) within a humidified H35 HypOxystation (Don Whitely Scientific) for a period of 2 or 4 h. All chemical compounds (Sigma-Aldrich) were re-suspended in either water or dimethyl sulfoxide (DMSO) and incubated at concentrations commonly used in the literature for a period of 1 h prior to exposure to A-body-inducing stimuli. Immediately following A-body-inducing treatments, cells were fixed with methanol, washed with 0.2 ng/mL Hoechst 33,342, and mounted on glass slides using Fluoromount G (ThermoFisher Scientific).

### Fluorescence microscopy, quantifications, and fluorescence recovery after photobleaching

Transfected cells were fixed with methanol (as described above), and probed with anti-CDC73 (Invitrogen, PA5-26189) antibody. Cells were then mounted on glass slides using Fluoromount G (ThermoFisher Scientific). Congo-red staining was carried out as described previously^27^. Briefly, following treatment cells were fixed in 4% formaldehyde, permeabilized with 0.5% Triton X-100, stained with a 1X (0.05%) Congo red solution, and mounted onto glass slides with 1% glycerol. Live-cells were stained with Amytracker 680 (Ebba Biotech) according to manufacturers instructions. Fluorescently-tagged and immunofluorescent, Congo red, or Amytracker stained cells were visualized on a Zeiss LSM880 laser scanning microscope with Airyscan and ZEN 2.3 software (Carl Zeiss Microscopy). For fluorescence quantification, images of 10 cells per biological replicate were captured. The percentage of cells with protein in the A-bodies was calculated by counting 100 cells and scoring each for the presence of A-body targeting. Relative A-body intensity was determined for treated samples to assess the effects of each compound on A-body recruitment as previously described^28^. Data was generated from at least 3 independent replicates. Fluorescence recovery after photobleaching (FRAP) experiments were carried out as described previously^27^. Briefly, cells were treated and visualized on a Zeiss LSM880 laser scanning microscope with Airyscan and ZEN 2.3 software (Carl Zeiss Microscopy). A-body sections, nuclear aggregates or sections of the nucleoplasm were bleached (100% argon laser at 488 nm) and monitored for the indicated time periods. Fluorescence intensity measurements were taken using ImageJ as described previously^67^. Data was generated from at least 10 cells per sample.

### Insoluble fractionation and western blotting

Insoluble fractions were collected as previously described^27–29^. Briefly, cells were washed in 1xPBS, prior to lysis with NP40 buffer (1% NP40, 50 mM Tris–HCl, 150 mM NaCl) and sonication (10% power for thirty 1s pulses). An aliquot was taken as the whole cell lysates (WCL), and the remaining sample was pelleted at 6010 g for 10 min. Pellets were then re-suspended in NP40 buffer by sonication (10% power for four 1s pulses). Western blotting was performed using anti-GFP (1:1000; Santa Cruz Biotechnology, sc-9996), anti-GAPDH (1:1000; Santa Cruz Biotechnology, sc-25778), anti-histone H3 (1:10,000; Cell Signaling, #9715), anti-COX1 (1:1000; Abcam, ab227513), anti-COX2 (1:1000; Abcam, ab151191), donkey anti-rabbit-IgG conjugated to HRP (1:10,000; Invitrogen, A16023), and donkey anti-mouse-IgG conjugated to HRP (1:10,000; Invitrogen, A16011) antibodies (Supplementary Fig. 1).

### Thioflavin T aggregation assay and dot blot

Thioflavin T (Sigma Aldrich) stocks were prepared by dissolving powdered dye in double distilled water, with 0.2 µm filtration done to exclude clumps. The β-amyloid (1–42) peptide (UltraPure, HFIP treated) used in this assay was a recombinant variant (rPeptide) that was suspended in 1% ammonium hydroxide to a final concentration of 200 µM. Experiments were carried out in 96-well plates (black chimney wells, with µClear bottom plates) and sealed with optical film. Each well contained Thioflavin T (10 µM), β-amyloid (1–42) peptide (10 µM), and the indicated concentration of the chemical compounds, with the final volume of the well adjusted to 100 µL using PBS (pH = 7.4). Chemical compounds were dissolved in DMSO, which was used as a vehicle control for the experiments. The different components of the reaction were individually pipetted into each well in the order: PBS, thioflavin T, experiment-specific compound, and the β-amyloid (1–42) peptide last. Mixing of the well components was carried out by gently pipetting the well solutions up and down, exactly 5 times. Thioflavin T was excited (450 nm) using a Synergy HTX microplate reader (Agilent) and emission (490 nm) fluorescence was measured every 2 min. The plates were incubated at 37 °C, and to enhance fibrillation rates and reproducibility^68,69^, we also shook samples for one minute between readings (60s of linear shaking with an amplitude of 1 mm and frequency of 886.9 RPM). Data was generated from at least 3 independent replicates. β-amyloid (1–42) (10 µM) was incubated with the indicated compounds in conditions identical to ones utilized for the Thioflavin T assay. After 2 h, 20 µL of the reaction mix was dotted on to a nitrocellulose membrane, and were allowed to air dry. The membrane was then probed using the anti-Amyloid Fibrils LOC (1:1000; Millipore Sigma, AB2287) and donkey anti-rabbit-IgG conjugated to HRP (1:10,000; Invitrogen, A16023).

### Statistical analyses

All graphs represent the mean values of at least three biological replicates. Error bars represent the standard error of the mean (SEM), and *p* values were calculated using the two-tailed Student’s t test, with the significance level indicated in figure legends.

### Supplementary Information

Supplementary Figure 1.

## Data Availability

All data generated and analysed during this study are included in this published article.
