# Supplementary material for: Stress-mediated aggregation of disease-associated proteins in amyloid bodies
Source: Sci Rep. 2023 Sep 2;13:14471. doi: 10.1038/s41598-023-41712-2 (PMC10475078; doi:10.1038/s41598-023-41712-2)
Supplement: Supplementary file 1 — Supplementary Figure 1. [file 41598_2023_41712_MOESM1_ESM.pdf]

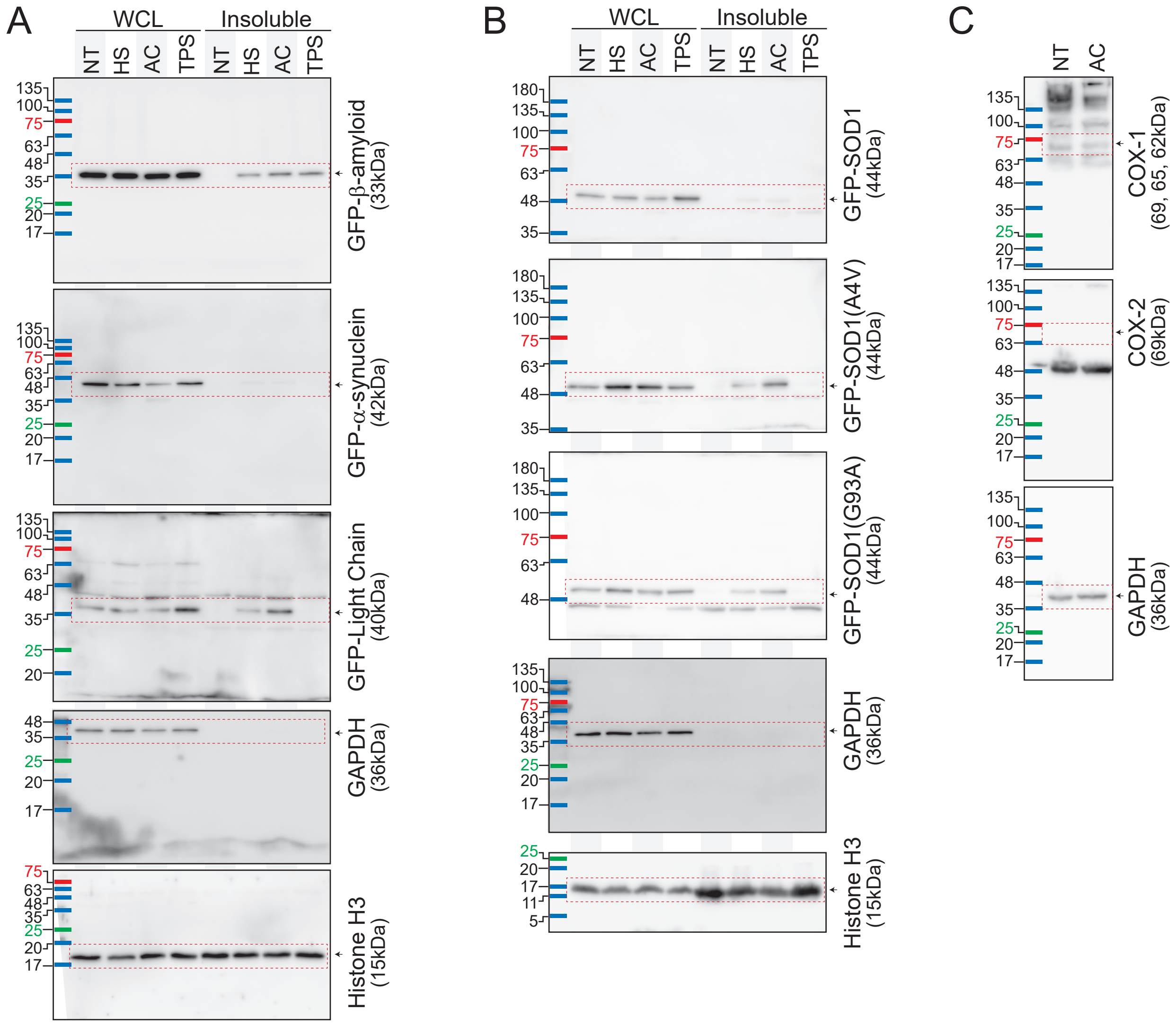

Supplementary Figure: Uncropped western blots. (A) Original blots for Figure 1C. The GAPDH and Histone H3 membranes were cut corizontally prior to hybridization. (B) Original blots for Figure 2G. The Histone H3 membrane was cut horizontally prior to hybridization. (C) Original blots for Figure 4D. All membranes were cut vertically, between wells, prior to hybridization. Red boxes indicate the cropped regions. Estimated protein sizes are presented (brackets-right).
